# A touchstone of Finnish curriculum thought and core curriculum for basic education: Reviewing the current situation and imagining the future

**DOI:** 10.1007/s11125-020-09533-7

**Published:** 2021-03-04

**Authors:** Liisa Hakala, Tiina Kujala

**Affiliations:** 1 Department of Education, Faculty of Educational Sciences, University of Helsinki, Siltavuorenpenger 10, P.O. Box 8, 00014 Univeristy of Helsinki, Finland; 2 Faculty of Education and Culture, Tampere University, Kalevantie 4, P.O. Box 607, 33014 Tampere, Finland

**Keywords:** Covid-19, Global crisis, *Bildung/Didaktik*, Curriculum, Curriculum design, Finland

## Abstract

Pandemics, like other global challenges, are unquestionably curricular issues. They are curriculum issues not only because of the disrupting consequences of Covid-19 and the economic and social crisis alike but also because people have, through their own activities, contributed to global catastrophes and perpetuated injustices. This article attempts to answer the question: How does Finnish curricular thought, including the role of the teacher and the core curriculum for basic education, respond to the various global crises? While reviewing the current situation, the article also imagines a post–Covid-19 curriculum. Reactivating what is still powerful in *Bildung/Didaktik* and emphasizing the importance of education’s ethical dimension and the teacher’s role as a curriculum theorist offer the means for dealing with the theme. In addition, understanding the structure of the National Core Curriculum document, the political dimension of the Finnish curriculum’s design process, and the educative possibilities in subjects and multidisciplinary modules, the teacher is capable of creating opportunities for educational experiences that are (ethically) significant for students, proactively and in terms of crises.

*In the midst of the coronavirus crisis, the heroes are those who keep the structures of society upright and the people alive. Teachers and early childhood educators are also part of this backbone of society*.

Helsingin Sanomat (2020a)

Over a short period, the global community has faced major challenges that require significant attention and remind us all of everyone’s global connectedness. These challenges include climate change, social inequality, the global refugee crisis, the threats to democracy—and the Covid-19 pandemic, which we are currently struggling with all over the world. The Covid-19 crisis, like other global challenges, is unquestionably a curricular issue. An accurate question is whether we can envision curriculum with the capacity to stay proactive and contribute to the prevention (or, at least, the management) of external threats and vulnerabilities, including outbreaks and emergencies. At the same time, global crises and challenges are touchstones for the prevailing curriculum tradition or thought and for the role of the teacher in this context. In this article, we ask: How does Finnish curricular thought, including the role of the teacher and the core curriculum for basic education, respond to the various global crises and challenges? While reviewing the current situation, we also imagine a post–Covid-19 curriculum.

## The Covid-19 pandemic challenges education and curricula

In January 2020, *Time* magazine devoted a special issue to young leaders. Its Editor-in-Chief, Edward Felsenthal, states in his editorial that the global under-30 population accounts for over half of the 7.5+ billion people on the planet. At the same time, young leaders raising their voices have become a force across the globe in such areas as climate, inequality, corruption, and freedom (Felsenthal 2020). Activist movements that drive change around the world are often the inspiration for this new generation of leaders, as Aryn Baker (2020), the Africa Bureau Chief for *Time*, notes. Among the interviewees of the special issue was the prime minister of Finland, Sanna Marin, who, since December 2019, has led a five-party coalition government with four other female leaders—all but one of them below the age of 35. Marin tells *Time* that she got into politics because she thought the older generation was not doing enough about the big issues of the future (Abend 2020). Climate activist Greta Thunberg and other young people involved in activist movements have constantly repeated the very same message: the efforts and actions of the older generations are inadequate in the face of current and future challenges.

*Time* published its special issue a few weeks before the World Health Organization (WHO) declared the novel coronavirus outbreak a public health emergency of international concern. It was evident very quickly that the toxic consequences of Covid-19 would not be limited to serious health threats. Covid-19, like other pandemics, has significant economic, social, and security impacts. A lesson learned from pandemics such as Ebola is that they reduce, if not ruin, the quality of life of communities and families, weaken economies, disrupt essential services (such as health, education, transportation, travel), and isolate people (Nabarro and Wannous 2016; see also Qiu et al. 2017). Additionally, as the Secretary-General of the United Nations (UN) António Guterres recalls, there are other far-reaching and challenging implications of pandemics such as Covid-19, including obstructed conflict resolution efforts, worsening of human rights situations, and growing manifestations of authoritarianism (UN 2020).

The Covid-19 pandemic and related protection measures have challenged education and curriculum drastically, in the short term and beyond. The temporary nationwide closure of educational institutions as a means of preventing the virus’ spread has affected more than 72% of the world’s students (UNESCO 2020a). School closures have numerous adverse consequences: the most severe are for the most vulnerable students and their families (UNESCO 2020b) and take different forms in different countries. Based on news reports, newspaper articles, and media debates, including social media sites maintained by teachers, Finland also had concerns linked to school closures. Teacher-led distance learning replaced contact teaching in basic education (grades 1–9) in Finland for approximately two months in the spring of 2020. However, schools were not totally closed. Pre-primary education organized in schools and contact teaching for pupils in grades 1–3 continued for the children whose parents work in sectors critical to the functioning of society. In addition, pupils with a special-support decision were provided contact teaching where necessary. The government’s recommendation was that, whenever possible, all children be cared for at home.

Although, in the Finnish media debates, there was a shared the view that teacher-led distance learning was generally working well, people raised concerns especially about underprivileged students who neither received support nor had the electronic devices or self-direction skills needed for distance learning. As one guardian writes: “A teenager with ADHD in distance learning. No way, will flunk the grade” (Helsingin Sanomat 2020b). In a survey addressed to members of Finland’s Trade Union of Education (OAJ), 63% of the respondents felt that the exceptional situation has negative consequences on the learning of basic-education students (OAJ 2020). (Note that, in the same survey, three-quarters of teachers reported that distance learning can also have positive effects on individual students, such as those having problems concentrating or who struggle with school anxiety, among others.) Respondents also perceived that, as a result of the lockdown, the ability to meet students’ fundamental needs was under threat. This was because of the students’ isolation from everyday social networks, potential substance abuse and/or domestic violence in some homes, and the lack of the usual free school meals. Many households that were already experiencing income difficulties had to resort to food aid (Soste 2020). Efforts were made to solve these problems. Some schools provided laptops, in some municipalities alternative measures were taken to secure the daily meal for those in need, and many teachers not only contacted their students daily but also were reachable online throughout the school day. Covid-19 highlighted the role of the school not only as an education provider but also as an important actor as an organizer of welfare services.

Despite all efforts, the Covid-19 pandemic has negatively affected the learning and well-being of some students, and the effects can be far-reaching. The economic impacts of Covid-19 on families cannot be ignored, either, as we learned in Finland recently from the great financial crisis of the 1990s. The results of a 25-year follow-up study of approximately 60,000 persons born in 1987 indicate that a family’s financial problems in a person’s childhood can result, later in life, in his/her mental health problems, in the lower grades at school, in shorter educational paths, and in difficulties coping independently financially (Ristikari et al. 2016). By April 2020, more than 300,000 people had received notice of dismissal or layoff due to the pandemic, or were already laid off or unemployed for the same reason. In addition, 186,000 were already unemployed or laid off before the pandemic (Pärnänen 2020). To date, 1 out of 10 Finns are included in these groups. At the same time, we are aware of the alarming situation of the poorest people in the world. According to the World Bank (2020), for the first time in 22 years, the poverty rates will rise. It is estimated that 40 to 60 million people will fall into extreme poverty (under $1.90/day) in 2020, due to Covid-19. We can hardly imagine the destructive consequences of increased inequality for individuals and societies, and for the global community.

Pandemics, like other global challenges, are unquestionably curricular issues. Not only the disrupting—if not devastating—consequences of Covid-19 and the crisis alike, but also that people have, through their own activities and imprudence (e.g., Foster, Salonen, and Keto 2019, pp. 123–24), contributed to global catastrophes and perpetuated injustices, unquestionably makes these things curricular issues. Moreover, perhaps more than ever, our endangered planet needs not only wise decision makers, but also educated citizens. Citizens who do not indulge in populism, despising democracy, who aspire for broader understanding, who consider the consequences of their own everyday choices and actions (as well as the perspective of the global community and of nature), and who are willing to act for a more just and sustainable future. Here, curriculum documents play an important role.

## Two major modes of Western curriculum thought

Scholars have employed two basic modes of thought in conceptualizing Western education, including its interrelated notions of teaching and learning: German-Scandinavian *Bildung/Didaktik* and Anglo-American *Curriculum*. This dichotomy does not do full justice to the theoretical, political, and practical underpinnings of education in different European countries. National characteristics may shape each, or countries may draw elements from both. A shift in emphasis from one model to another may also occur, as is the case with Finland; this happens without completely giving up either model, however (Autio 2006). Here, we focus on these two approaches, which Westbury (1998) describes as “very different intellectual systems” that “seek to do very different kinds of intellectual and practical work” (p. 48).

Bildung, having its roots in German philosophy, began to establish itself in educational discourse in eighteenth-century Germany. (Bildung is often translated as “formation” or “cultivation”.) It is foremost a theory of becoming human (Autio 2014; Horlacher 2017). The ideal aim of Bildung is an autonomous, self-determined, and self-reflected personality (Schneider 2012, in Horlacher 2017, p. 1). In the approach, one presumes a connection between an individual’s inner cultivation (an idea of individual perfectibility resulting from continuous study and other activities) and the development of a better and/or more just society (Autio 2014, p. 18; Horlacher 2017, pp. 2, 103–106). A person who is capable of understanding, of interpreting, of justifying, and of criticizing collective “truths” is a person capable of intelligent social action (Stojanov 2012). To pursue these ideals, educational institutions should have relative autonomy in relation to the rest of society (Autio 2014, 2019). In addition, teachers should approach the moral requirement of treating their students and the processes of Bildung as ends in themselves. In this framework, it would be immoral to see students only as bearers of human capital. It would be equally immoral if educational institutions considered the maintenance of any particular traditions to be their main task (Stojanov 2012).

*Didaktik* denotes the view of curriculum theory as closely intertwined with Bildung (Bildung/Didaktik), yet emphasizing classroom curriculum (Autio 2014; Westbury 1998). Here, the philosophical and the theoretical dimensions of education are seamlessly connected to the practical dimension of teaching, and this occurs through the work of the teacher. Teachers are curriculum theorists or professional practitioners who have the expertise needed for the realization of *Bildungsideal* when teaching their students (Westbury 1998, pp. 51, 57). It is the task of universities and higher administration to select and organize the subject matter “worthy of educational processes” presented in *Lehrplan*—a teaching plan or curriculum (Künzli 1998, p. 32). Yet, the state-mandated and subject-based *Lehrplan* does not just straightforwardly dictate teachers’ work. The tradition emphasizes the teacher’s freedom to teach: it is the teacher’s task to interpret the curriculum, to explore it and its potential to be educative. The content to be taught is educative when it has broader (ethical) significance for the life of the specific group of students, now and in the future (Künzli 1998; Westbury 1998). It is exactly for these reasons that the curriculum can never be totally predetermined and fixed, nor can the knowledge addressed in classrooms be static, something to be just delivered—with tests in sight. Both the teachers and the students should always have opportunities to reflect on the worthwhileness of the subject matter (Autio 2014, p. 18).

Anglo-American *Curriculum*, in turn, is widespread curriculum theory, with its roots in the late-nineteenth- and early-twentieth-century United States. Since the early days of the tradition, administrative interest in the practice has been primary (Pinar et al. 1995). In the early twentieth century, education was expected to meet the new demands of an industrializing society. Maintaining social order and promoting economic growth became the main tasks of education (Kliebard 1999; Labaree 2010; Tröhler 2011). Together, experimental psychology, promising empirically verified and objective truths; and scientific management, a theory connected to working life, formed the new scientific basis for education. Social efficiency, supported by administration and furthered by this new scientific basis became an enduring catchword of education. Curriculum figuratively became an assembly line producing citizens who were economically and socially useful according to the perceived needs of each period. This utilitarian and rational view of education—emphasizing standardization and, later, also accountability—still dominates Anglo-American curriculum (Autio 2017; Labaree 2010; Pinar et al. 1995; Westbury 1998). Curriculum theorists have strongly criticized it, however (see Pinar et al. 1995).

Since 1995, Pinar has offered a closer articulation of his intellectual stand on Bildung: “One hundred years ago, Americans travelled to Germany … to study concepts of education. It seems to me it is time again to selectively incorporate German concepts in North American practices of education” (Pinar 2011, p. xiv). Pinar’s motivation to “selectively incorporate” German concepts is to engage curriculum theory in the political cause of *democratization*. He does so by introducing the concept of *currere:* transforming the traditional excessively apolitical, aesthetic, and theological emphases of Bildung, and disclosing the assumedly neutral, objective, and psychologized US curriculum as a political, yet not necessarily democratic, construct:

Despite its displacement in some countries by traditional US curriculum theory, in recent years, *Bildung* has enjoyed something of a revival, thanks in part *due to its wedding with democratization*…*Without Bildung*, Karsten Schnack…asserts, *democracy is an “empty shell, a procedure or form of government*”. Commitment to inner development and social democracy are juxtaposed in my conception of curriculum as lived experience: *currere*. (Pinar 2011, pp. 4–5; emphases added)

In the same intellectual and political fashion, Wolfgang Klafki in Germany, in his critical-constructive Didaktik, sought to articulate the progressive democratic potential of Bildung by incorporating the Frankfurt School programme into his theory (Klafki 1998). Today, his theory comprises a comprehensive democratic argument—relating to topics from classroom activities to policy measures—in debates with current neoliberal education and curriculum reforms. As Ryen (2020, p. 227) states:

While the increasingly abstract and formalistic curricula can be seen as an attempt to provide education that enables students to face an uncertain and ever-changing future, replacing the content with competency is bound to fail (Willbergh 2015). The strength of critical-constructive Didaktik is that it enables educators not only to criticize such policy trends but also provides a powerful tool for selecting and working with content in concrete classroom settings. The epistemology it offers could, therefore, be attractive to curriculum scholars who seek to…challenge the positions of measurement specialists, learning scientists and educational technologists (Deng 2018). I believe this is not only a pressing pedagogical task but a democratic one, which makes the continued engagement and dialogue between Didaktik and curriculum studies all the more important.

In the Curriculum approach, the role of the teacher differs from that in the Bildung/Didaktik approach. In the former, there is much less institutional trust in teachers’ professionalism. The teachers’ task is not to interpret but to implement the curriculum (Westbury 1998). As one teacher-educator from the US sums up in an email discussion in 2019: “In our state, everyone is supposed to use the state standards to guide them, but there are so many programs for sale on how to reach the best results for kids. Schools will pay for reading programs, math programs etc. to use as a canned curriculum that all teachers have to follow”. In curriculum, much attention is paid to support materials for teachers; in Bildung/Didaktik, most resources are directed to teacher education (Erss 2017, p. 200).

Finnish curriculum tradition is, historically, a mixture of influences from the two curriculum approaches above. The ideal of Bildung still prevailed in Finland in the first half of the twentieth century. After World War II, Finland adopted ideas about education not from Germany but from the United States. Rational planning and behavioral (later, cognitive) psychology entered the field of education, and the spirit of Bildung inherited from German philosophy and science of education became marginalized. Instead of giving up teacher autonomy, it gained a new, scientific basis (Saari, Salmela, and Vilkkilä 2014); the idea of the teacher as a researcher gradually emerged. This scientific basis fostered by research-based teacher education legitimized teacher autonomy, and still does. However, as Sitomaniemi-San (2017) points out, methodology—not theory—has driven the work on research-based teacher education, thus limiting the opportunities for teacher-students to deepen their understanding of the complexity of curriculum.

Today, as stated, our endangered planet needs not only wise decision-makers but also educated citizens and subjects. In our view, this requires reactivation of some ideas of Bildung/Didaktik. It requires autonomous teachers who aspire to broader understanding, who are thoughtful, critical, and ethically committed, and who are capable of understanding and interpreting the curriculum, and of exploring it and its potential to be educative.

## Finnish curriculum design and curriculum documents

In the 1970s, Finland established a major structural educational change: comprehensive school reform. It replaced its dual, segregating, and thus unequal, education system with a uniform, comprehensive school system—basic education (grades 1–9) and its curriculum (Ahonen 2003). Since then, it has renewed the National Core Curriculum for Basic Education (NCC) approximately every 10 years. Ministry-level administration coordinates this process, which follows a regular bureaucratic cycle. Traditionally, the Finnish curriculum is subject-centered, in the spirit of Bildung/Didaktik. The government determines the general national education objectives and the distribution of lesson hours for different subjects. The Finnish National Agency for Education (FNAE), working with the Ministry of Education and Culture, determines the objectives and core contents of subjects and cross-curricular themes. The NCC 1970 was the last document implemented locally as such (National Board of Education 1970). Today, the local curriculum—the outcome of an integrative curriculum process conducted with local education authorities and schools—is a significant part of the curriculum design process. The purpose of the local curriculum is to set out and implement national targets and goals, and tasks considered important locally (FNAE 2014; Vitikka and Rissanen 2019).

### The structure of the NCC 2014

The latest curriculum, NCC 2014, came into effect in August 2016. It is divided into two parts: one is general, identifying the ultimate focal points of education. The other part describes the mission, objectives, core contents, and forms of evaluation for individual school subjects. According to the NCC 2014, students acquire competence in individual fields of knowledge and in themes that cross subject boundaries. The latter includes:

1. transversal competencies, such as: thinking and learning to learn; cultural competence, interaction, and self-expression; and participation, involvement, and building a sustainable future; and
2. multidisciplinary learning modules, meaning: themes in keeping with the principles of the school culture, interesting to pupils, and suitable for cooperation not only between subjects and teachers but also between the school and the society around it. (FNAE 2014, pp. 21–26, 32–34)

Gradually, the structure of the curriculum document has shifted from subject content to general objectives. Unlike the 2004 National Core Curriculum, which still emphasized subject content, the NCC 2014 emphasizes general objectives in terms of key competences as mentioned above. To ensure the status of the transversal competencies, they have been linked in the definition of the subjects’ objectives and key content areas (FNAE 2014, p. 21). Therefore, the NCC 2014 very clearly indicates the links between the competencies and the subjects. Interestingly, transversal competencies reflect, in part, a Europeanization process, as they correspond to the eight key competencies that the European Union advanced. This move toward emphasizing general objectives instead of subject-matter teaching is a fairly dramatic shift in the Finnish education system (Hardy and Uljens 2018, pp. 63–64).

Multidisciplinary learning modules represent an integrative approach to teaching—that is, teaching aimed at educating integrative thinkers with interdisciplinary skills. Teachers guide students toward understanding relations between the same topics in different school subjects (Haapaniemi et al. 2020). Emphasizing multidisciplinary skills and cross-curricular themes in curricula, grows, however, from the critique of the subjects’ contents and those contents’ability to provide students with future skills (see Halinen and Jääskeläinen 2015, p. 27). This view of subject content ignores both the personal growth highlighted in Bildung/Didaktik and the moral, intellectual, and cognitive resources one is able to reach with profound familiarization with the subjects. These qualities cannot be reached through an instrumental skill- and competence-based curriculum (Autio 2019). In this framework, it makes sense that teachers express concern about their ignorance of subject-specific ideas (Haapaniemi et al. 2020). However, teachers can use multidisciplinary learning modules very well to address timely topics such as pandemics and related factors. Thus, we should not see multidisciplinary learning modules and school subjects as mutually exclusive. With both, it is, instead, a question of their educative use.

### The curriculum design process as interactive and inclusive

Policymakers in Finland intend the curricular process to be interactive and inclusive, carried out in collaboration with teacher-educators and -researchers, educational providers, schools, and other interest groups identified as important actors in the field, such as pupils and their guardians. There is also an open online platform for all citizens to participate. The FNAE provides official information that emphasize the process’s interactivity and inclusivity. However, we must take a closer look at that process.

Various interest groups are involved in the process. The Association of Finnish Municipalities, Working Life (SAK), Confederation of Finnish Industries (EK), the Trade Union of Education in Finland (OAJ), political parties, and a variety of think tanks try to impact the political agenda in education policy to strengthen the position of their members (Tervasmäki and Tomperi 2018). These groups organize invitational seminars and hearings with invited stakeholders; 60,000 young people participated in web interviews (Lähdeniemi and Jauhiainen 2010; see also Hakala 2011). In this sense, one can view the process as interactive and inclusive; moreover, the process is to a great extent political, and involves a variety of interest groups and contradictory views. However, when comparing the web interviews and the open online comments to the final version of the curriculum document, they did not play a significant role (Lähdeniemi and Jauhiainen 2010; Säily et al. 2020); of particular concern is that the process may not have given a sufficient hearing to the voices of young people. It appears that FNAE, accompanied by global neoliberal influencers (Autio 2017; Saari, Salmela, and Vilkkilä 2014; Tervasmäki and Tomperi 2018), dominates the NCC design process, with strong control over the final curriculum. In these senses, the democratization of the curriculum design process has not succeeded as it should have.

In the same regard, one must also question the local process. Although cooperation, for example, with pupils’ guardians is officially encouraged and it is possible for pupils and their guardians—in the person of school governors—to be involved, principals and other municipal officials seem to control the process (see Vitikka and Rissanen 2019). It appears that the education bureaucracy has formally implemented the idea of an interactive and inclusive curriculum design process, but the democracy of that process is not quite established, even at the local level.

Curriculum design processes are no longer limited simply to the individual nation-state. To an increasing degree, they reflect transnational influences. For example, OECD and UNESCO are remarkable key actors in education policy. Standardized student assessment, test-based accountability, technology-assisted teaching and learning, and proficiency in basic skills (e.g., reading, mathematical, and scientific literacy) have become common priorities in education reforms around the world since the 1980s. Finland has not adopted these Anglo-American curriculum-oriented elements in the same way that many other countries have (Autio 2017; Sahlberg 2011). However, there certainly is pressure to accept these market-driven education reforms that have landed in Finland. Global competitiveness was the priority of the previous Finnish government (from 2015 to 2019). Accordingly, the main objectives of education policy were modernizing and digitalizing the learning environments, and strengthening the relationship between education and work life. Thus, commercialization of the schools was accepted despite the NCC’s statement that “the school and education may not be used as channels of commercial influence” (Tervasmäki and Tomperi 2018). In the name of digitalizing education, computer, software, and license providers spoke out on pedagogy—acquiring quite a lot of influence not only on education policy in general but also in individual schools.

To sum up, the Finnish curriculum design process is intended to be interactive and inclusive. However, the FNAE—accompanied by global neoliberal influencers—simultaneously struggling against the spread of market-driven education reforms, dominates the NCC design process and has strong control over the final curriculum. An Anglo-American curriculum-related, goal-oriented, and fixed curriculum is a risk in a situation like that. Yet, as Autio (2017) states, the Finnish education system has succeeded in remaining surprisingly immune to those powerful political and transnational economic forces that, in many parts of the world, have driven basic education and teachers’ work in the worsening education crisis. This can be explained by the Programme for International Student Assessment (PISA)’s success in proving the strength of Finnish curricular thought, by academic teacher education, and by the culture of trust in teachers’ professionalism (Autio 2017, p. 44). Bildung/Didaktik, still vital in Finland, emphasizes teachers’ freedom to teach and to interpret curriculum. Exploring the curriculum and its potential to be educative can also mean broadening and adjusting the current curriculum to address the specificities of students’ situations.

## Analyzing the Finnish national curricula from the teacher’s point of view

To interpret the curriculum and to explore it and its educative potential, the teacher must be aware of the curriculum design process (the multiple influencers participating in it) and of the historical curriculum chain (the shift from one curriculum to another). In addition, they should scrutinize and challenge the “truths” of individual school subjects.

As an example, we present in Table 1, below, the analysis of four consecutive Finnish curricula documents (FNAE 1985, 1994, 2004, 2014) for grades 1–9 (years 7 to 15; Hakala and Kujala 2015, 2017). The analysis focuses on the aims of the general part of the curricula and the relation of one school subject (physical education, or PE) to them. The aim being addressed is considered educative when it has broader (ethical) significance for students’ life worlds, in the present moment and in terms of their future (Künzli 1998; Westbury 1998).

**Table 1:** Summary of the aims of education in general part and in PE in the four most recent Finnish national core curricula for basic education

The aims of education	FNAE (1985)	FNAE (1994)	FNAE (2004)	FNAE (2014)
Global education	Global education for international understanding, co-operation and peace (p. 13)	Internationalization of operating environments, e.g. the endorsement of multiculturalism and European integration (p. 14)	Endorsement of multiculturalism, social, and cultural diversity, **enhancing tolerance** and intercultural understanding as underlying values of basic education (pp. 36–37)	**Establishing the basis of global citizenship, promoting cultural diversity as a positive resource** (pp. 16, 21)
Cultural education	**Common cultural heritage and independent home country as a basis of national identity** (p. 12)	**National cultural heritage** linked with other Nordic countries as a basis of identity (pp. 13–14)	**Formation of the pupil´s own cultural identity, and his or her part in Finnish society** and in globalizing world (p. 12)	**Cultural interaction. Formation of the pupil´s own cultural identity, awakening the interest in other cultures, promotion of cultural know-how, appreciation of cultural heritage and the democratic values in Finnish society** (pp. 16–19)
Education to gender equality and diversity	Gender equality in family life, working life, and society (p. 13)	Gender equality in family life, working life, and society (p. 14)	Gender equality in family life, working life, and society (p. 12)	**Equality, parity, and social justice. Promoting equal opportunities, deconstructing traditions and stereotypes concerning gender, acknowledging gender diversity, supporting gender identity and sexual identity construction** (pp. 14–18)
Ecological education	Nature conservation as a part of the environmental education (p.12)	Sustainability from justice point of view (p. 13)	Natural diversity and preservation of environmental viability as underlying values of basic education (p. 12)	Sustainability (ecological, economic, **social**, and **cultural**), eco-social education, and global responsibility. School and education should not be used for promoting commercial purposes (pp. 15–16)

*Source:* Hakala and Kujala (2015, 2017)

*Note:* Italics – excluded in PE, bold – included in PE

In Table 1, the curricula analysis reveals that PE is committed to the idea of a homogenous Finnish society and culture. Until the NCC 1985, PE content was separate for girls and boys; even today, PE classes are mainly single sex at the secondary level, although there is no legal basis for this. The NCC 2004, in its PE curriculum, adopted a slight shift towards intercultural emphasis, in terms of global education. The question of gender seems to have been unproblematic on the whole, and traditional gender roles were a given in the PE curricula until NCC 2014. Surprisingly, although outdoor education is an important part of PE, the PE curricula almost completely excludes ecological aims. Unlike in the general part, the PE curricula seems to lag behind in the development of interculturality, globalization, and ecological issues.

To find an explanation, the teacher must look back to history and to the prominent role of sports’ constructing not only the nation-state and nation-states’ citizens in Finland but also the subject of PE. When looking more closely at the history of PE, we see that nationalism, given gender roles, rationality, self-discipline, and achievement orientation, among other things, have been ingredients of sports (Dunning 1993; Korsgaard 1982; Mangan 2000). We have suggested that these issues restrain alternative discourses in PE curricula even today and, simultaneously, not only alienate students from their bodies but also prevent their self-understanding and their growth in moral subjects (Hakala and Kujala 2015, 2017).

To encounter students’ life worlds, it is essential for the teacher to understand the historical foundations as well as the current unethical discourses influencing PE. When understanding the burdens of the subject, the teacher and students, as well, are capable of thinking against the subject matter (Autio 2014, p. 18) and together producing more ethically responsible PE. Having the ethical dimension as a starting point, a school subject can serve as a platform for dealing with all timely topics, even the most difficult ones.

## Conclusions

In this article, we asked: How does Finnish curricular thought, including the role of the teacher and the core curriculum for basic education, respond to various global crises and challenges? While reviewing the current situation, we also imagined a post–Covid-19 curriculum. Our starting point was that German-Scandinavian curricular thought offers an alternative way of “thinking curriculum”. Reactivating what is still powerful in Bildung/Didaktik not only acts as a counterweight to the widespread utilitarian ideas of curriculum but also provides means for dealing with the unpredictable future, including global crises and challenges, in an ethical manner. Although Bildung lost its dominant position in Finnish curricular thinking after World War II, one can easily find the ideas in Finnish education, including curriculum documents that even today demonstrate their vitality and show the impossibility of totally breaking the ties with history and culture. For example, in its chapter “Underlying Values of Basic Education”, the NCC 2014 states: “Each pupil has the right to grow into his or her full potential as a human being and a member of society”; “Basic education promotes well-being, democracy and active agency in civil society” (FNAE 2014, pp. 15–16). There is also emphasis on broad knowledge in Finnish curricula. Instead of emphasizing basic skills, equal value is given to all aspects of individual growth; that is, to personality, morality, creativity, knowledge, and skills.

In the spirit of Bildung, there is a culture of trust in teachers. According to the Global Teacher Status Index 2018, 9 out of 10 Finns show confidence in teachers. Teachers are considered hardworking, intelligent, and caring (Dolton, Marcenaro, De Vries, and She 2018). “Caring” refers to the teacher-student relationship, representing that of Bildung/Didaktik. This pedagogical relationship is personal, being guided by teachers’ professional ethics (see Künzli 1998, p. 36). Teachers’ professional ethics were also at stake when schools closed due to the Covid-19 pandemic. In mid-March 2020, a state of emergency was declared in Finland as a result of the coronavirus outbreak. All school premises closed their doors, and contact teaching was suspended. Within a few days, Finnish teachers, who had been patronized for years because of their alleged incompetence in digital literacy—the Confederation of Finnish Industries and the 2015–2019 right-wing government at the forefront of these attacks (Tervasmäki and Tomperi 2018)—launched distance teaching at all class levels. Teachers performed this task responsibly for two months. Indeed, many factors seem to indicate that Finnish curricular thought and the NCC 2014 are capable of responding quite well to the management, at least, of external vulnerabilities such as Covid-19. However, based on our review, we suggest paying more attention to the political dimension of curriculum and to the role of the teacher as a curriculum theorist, in order to bring about a post–Covid-19 curriculum that is more conscientious and proactive.

In Bildung/Didaktik, there is a moral requirement of treating students and the processes of Bildung as ends in themselves. When understanding the Finnish design process of the curriculum, the structure of the curriculum documents, and the educative possibilities of subjects and multidisciplinary modules, the teacher is capable of creating opportunities for educational experiences that are (ethically) significant for students and have a solid connection to the world around them.

Understanding the political character of the curriculum design process and its multidimensionality is essential. The newest curriculum process, for NCC 2014, was formally implemented as interactive and inclusive. However, research reveals that the process was not entirely democratic. We consider this to be symptomatic. In the future, more attention should be paid to truly democratizing the curriculum process. It is particularly worrisome that more powerful stakeholders overshadowed the voices of the generations whose future is at stake. Obviously, the voices of the students should be heard in everyday work at school, too; students should have opportunities to deal with ethical dilemmas, to identify alternative course for action, and to practice argumentation on an ethical basis in every school subject.

Being aware of the history of education of the subject at issue, including the intertwined “truths”, the teacher can offer students opportunities to deconstruct and reconstruct their self-understanding. For example, by highlighting in PE the narrow image of masculinity offered by sports, the teacher creates space for a wider range of masculinities. Equally, when teaching, the teacher can and should address themes such as equality and equity, social injustice, and ecological issues, among others. It is also important to build mutual trust (the value of which is emphasized during crises) and a sense of connection with all forms of life. In our view, teaching like this is in line with the critical-constructive didactics of Klafki (1998, pp. 311–14), referred to earlier. (Interestingly, Wolfgang Klafki sees a connection between his critical-constructive Didaktik and William Pinar’s thoughts of curriculum studies [Klafki 1998, p. 327].) Such a teaching, where it is possible to construct both one’s self-understanding and worldview, is particularly relevant at a time when we are trying to respond to fast-changing and unpredictable crises such as Covid-19.

As stated, our endangered planet needs not only wise decisions-makers but also educated citizens and subjects. It requires autonomous teachers who are free to teach according to their professional intellectual judgment (see Pinar 2012, pp. xvii–xviii). Such an understanding of the teacher, innate in Bildung/Didaktik, is something to be cherished. It can also be seen as a necessary condition for imagining a curriculum that provides opportunities for students to understand what is happening to their parents, to their societies, to them:

The point of public education is to become an individual, a citizen, a human subject engaged with intelligence and passion in the problems and pleasures of his or her life, problems and pleasures bound up with the problems and pleasures of everyone else in the nation, everyone on this planet. (Pinar 2012, p. 229)
